# Epigenomic insight of lingonberry and health-promoting traits during micropropagation

**DOI:** 10.1038/s41598-022-16530-7

**Published:** 2022-07-21

**Authors:** Arindam Sikdar, Umanath Sharma, Rajesh Barua, Abir U. Igamberdiev, Samir C. Debnath

**Affiliations:** 1 St. John’s Research and Development Centre, Agriculture and Agri-Food Canada, St. John’s, Newfoundland and Labrador Canada; 2 Department of Biology, Memorial University of Newfoundland, St. John’s, Newfoundland and Labrador Canada

**Keywords:** Biological techniques, Molecular biology, Plant sciences

## Abstract

Epigenetic variation plays a role in developmental gene regulation and responses to the environment. An efficient interaction of zeatin-induced cytosine methylation and secondary compounds has been displayed for the first time in tissue-culture shoots/plants of lingonberry (*Vaccinium vitis-idaea* L.) cultivar Erntedank in vitro (NC1, in a liquid medium; NC2, on a semi-solid medium), ex vitro (NC3, node culture-derived plants; LC1, leaf culture-derived plants) and its cutting-propagated (ED) plants. Through methylation-sensitive amplification polymorphism (MSAP) assay, we observed highest methylated sites in leaf regenerants (LC1) from all primer combinations (108 bands), along with the highest secondary metabolites. The four types of tissue culture-derived shoots/plants (NC1, NC2, NC3, LC1) showed higher methylation bands than cutting propagated donor plants (ED) that exhibited 79 bands of methylation, which is comparatively low. Our study showed more methylation in micropropagated shoots/plants than those derived from ED plants. On the contrary, we observed higher secondary metabolites in ED plants but comparatively less in micropropagated shoots (NC1, NC2) and plants (NC3, LC1).

## Introduction

Lingonberry (*Vaccinium vitis-idaea* L.), a small perennial shrub, belongs to the genus *Vaccinium* L. of the Ericaceae family (subfamily: Vaccinioideae), which contains about 4250 species in 124 genera^1^. It is used for the production of jams, jellies and candies. It is widely spread through Greenland, Iceland, North America, Scandinavia, Northern Asia, and some other parts of Europe and Asia^2^ (supplementary Fig. S1).

Micropropagation is a quick and more efficient method to propagate lingonberries in masses that makes it possible to be done all the year through axillary bud proliferation and differentiation to mature plants formed from meristematic tissues to fully grown plants^3^. This process is called micropropagation or in vitro propagation. Lingonberry contains abundant secondary metabolites, including phenolic contents, flavonoids, and proanthocyanidin^4^. Young leaves may contain up to 1,740 mg/kg anthocyanin (fresh weight)^5,6^ along with 58% of total organic biomass phenolic content present in leaves, 48% in stems, and 79% in fruits^7^. Lingonberry has been found a high amount of antioxidant and antimicrobial activity in the fruit^8^. In contrast, flavonoid content exists between 27%–42% in leaf tissues^9^. It has been introduced as fruit from an ancient era and a medicinal plant and used as an ornamental plant for the landscape ecology^10^. Furthermore, leaf and fruit parts can reduce cholesterol levels, prevent rheumatic diseases, hepatitis C, kidney, bladder infections, and have been used to treat Alzheimer's disease^11,12^. Lingonberry fruits can be consumed raw and used to make juices, wines, pastries, sauces, jams, jellies, ice creams, cocktails and desserts^13^.

Epigenetic variation means DNA methylation and the modifications of amino acids as well as the tail of histones in the way of mitotically and/or meiotically heritable and non-heritable alterations^14^. Changes in the DNA methylation (or hydroxymethylation), histone modification or both are the crucial factors for epigenetic changes in in vitro plants^15^. The two types of DNA methylation exist active DNA methylation (regulated by gene expression) and passive DNA methylation (regulated by gene regulation)^16^. On the other hand, DNA demethylation refers to the removal of methylated group from the DNA. It can occur as an active DNA demethylation (enzymatic removal of methylated cytosine) or passive DNA demethylation (removal of DNA methylation during DNA replication)^17,18^. Waddington initially coined the term ‘epigenetic.’ A methyl group can be briefly incorporated in the fifth position of cytosine residues where plants have three apparent phases of cytosine: CG, CHG, and CHH (where H is C, A, or T)^19^. CHH methylation is distributed randomly across chromosomes, whereas CG and CH methylation have no genome-wide significance. When average CHH methylation of large transposons was incorporated in the chromosome, it represented more significant^20^. CG and CHG are regulated by DNA METHYLTRANSFERASE 1 (MET1), CHROMOMETHYLASE 3 (CMT3), and DOMAINS REARRANGED METHYLTRANSFERASE 2 (DRM2), which help in the catalysis of de novo DNA methylation. This mechanism is called RdDM. It was induced by DDM1 and CHROMOMETHYLASE 2 (CMT2)^21,22^.

Epigenetics studies have a major impact on agriculture due to the food supply and the consequences of global changes. Furthermore, it is essential to analyze the basic epigenetic mechanism in in vitro-cultured plant production. As the tissue culture plant tends to a wide range of epigenetic variation, it is possible to analyze breeding programs to establish a more diverse cultivar. Several studies reported that DNA methylation pattern stability was observed between in vitro and *ex vitro*-grown plants^23^. The epigenetic variation imprints the developmental program's memory^24^. In this way, we can get elite individuals without transgenic line generation^23^.

In epigenetic variation, similarities are found in the DNA sequence, but main changes occur in cytosine methylation^25^. There are various methods for DNA methylation analysis, including methylation-sensitive amplification polymorphism (MSAP), bisulphite sequencing, DArTseqMET, metAFLP and MethylRAD^26–28^. MSAP is the advanced form of AFLP, based on the sensitivity of restriction endonucleases to site-specific methylation^29,30^. It involves digestion with methylation-sensitive restriction endonucleases proceeded by amplification of restriction fragments. We report here, for the first time, the zeatin-induced cytosine methylation and its effects on secondary compounds in tissue-culture shoots/plants of lingonberry cultured in vitro and *ex vitro* conditions. The research results will be of significant importance for providing in-depth knowledge of the epigenetic regulation of secondary metabolism and bringing forth the key data on epigenetics for using them in the genetic enhancement program of *Vaccinium* species.

The present study aims to identify the differences in DNA methylation, and phenotypic and phytochemical changes of micropropagated shoots and plants with those of cutting propagated plants in lingonberry.

## Results

### Morphological pattern of in vitro and ex vitro of lingonberry

In this study, morphological data of node culture derived in vitro-grown shoots were compared between liquid (NC1) and semi-solid (NC2) media, and between node (NC3) and leaf culture-derived (LC1) tissue culture plants along with terminal softwood cuttings propagated (ED) greenhouse-grown plants of lingonberry cultivar Erntedank. In two node culture derived cultures, NC2 produced more vigorous shoots per explant (55.2 ± 2.05) and leaf number per shoot (16.2 ± 1.30) compared to NC1 (shoot number per explant: 42.8 ± 6.76; leaf number per shoot: 10.4 ± 7.13). The NC1 produced a greater shoot size (9.7 ± 2.31 mm) compared to NC2 (8.28 ± 1.17 mm). The shoot vigour of NC2 (rank: 4.80 ± 0.45) was better than NC1 (rank: 4.80 ± 0.84) in this study. The greenhouse-grown ED plants yielded a more vigorous plant with fewer shoots and leaf per plant compared to NC3 and LC1 plants. LC1 plants produced the highest number of rhizomes per plant (71.6 ± 4.28) and shoot number per plant (74.6 ± 4.28), while NC3 plants produced a comparatively lower number of rhizomes per plant (40.4 ± 4.10) and shoot number per plant (42.2 ± 2.17). ED plants produced lowest number of rhizomes (8.2 ± 0.84) and shoots per plant (11.2 ± 1.30). LC1 plants had longest rhizomes (10.8 ± 3.12 cm). For the number of leaf per shoot, softwood cutting ED was the best (24.4 ± 12.3) in comparison to NC3 (12.4 ± 2.07) and LC1 (14.6 ± 2.51). Additionally, the length of the shoot in LC1 was the highest (10.38 ± 4.52 cm) followed by NC3 (10.1 ± 2.48 cm) whereas ED represented the lowest length of the shoot (8.1 ± 3.38 cm). The length and breadth of the leaf appeared highest in ED plants (length: 2.34 ± 0.18 mm; breadth: 1.2 ± 0.24 mm) compared to NC3 (length: 1.36 ± 0.114 mm; breadth: 0.74 ± 0.114 mm) and LC1 (length: 1.5 ± 0.406 mm; breadth: 0.88 ± 0.192 mm). Also, the plant vigour was the best in leaf culture derived plants LC1 (rank: 8.0 ± 0.0).

### Recognition of cytosine methylation and its polymorphism using methylation-sensitive amplification polymorphism (MSAP) assay

**Figure 1:**
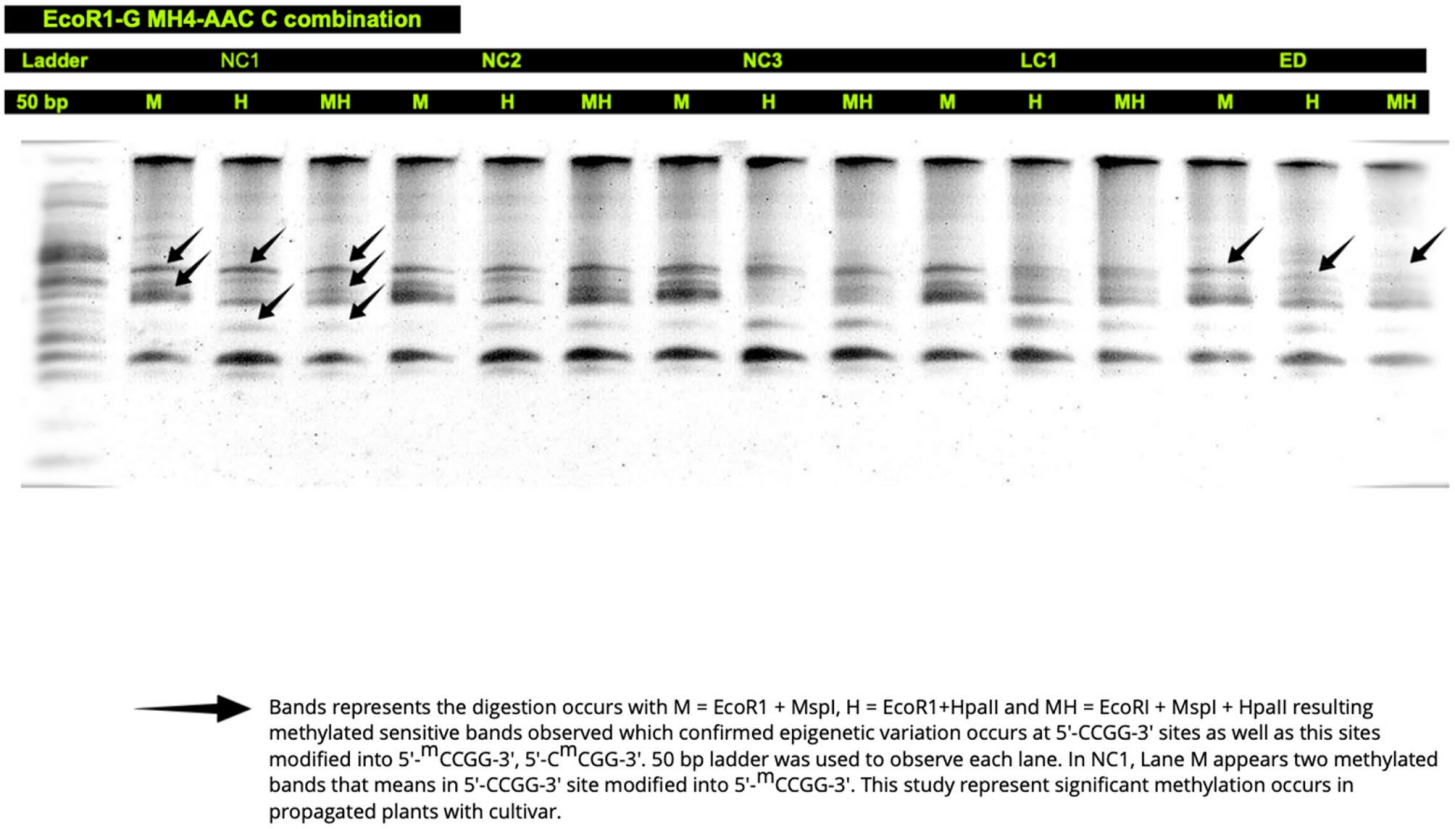
DNA methylation patterns observed in NC1 (node culture-derived shoots from liquid medium), NC2 (node culture-derived shoots from semi-solid medium), NC3 (node culture-derived plants from greenhouse), LC1 (leaf culture-derived plants from greenhouse), and ED (cutting propagated plants from greenhouse) of lingonberry cultivar Erntedank. Selective amplification was carried out using an *EcoR1*-G/MH4-AAC C primer combination. M, H, and MH refer to DNA fragments originating from digestion with combinations of *EcoR1* + *MspI*, *EcoR1* + *HpaII*, and *EcoR1* + *MspI* + *HpaII*, respectively. DNA bands (marked arrows) present in H digestion lanes but not in M lanes indicate hemi-methylated external cytosine (5'-mCCGG-3') at 5'-CCGG-3' sites, whereas DNA bands (marked arrows) present in M digestion lanes but not in H lanes indicate fully methylated internal cytosine (5'-CmCGG-3') at 5'-CCGG-3' sites in genomic DNA. Ladder: 50 bp (New England Biolabs Ltd., Whitby, ON).

**Figure 2:**
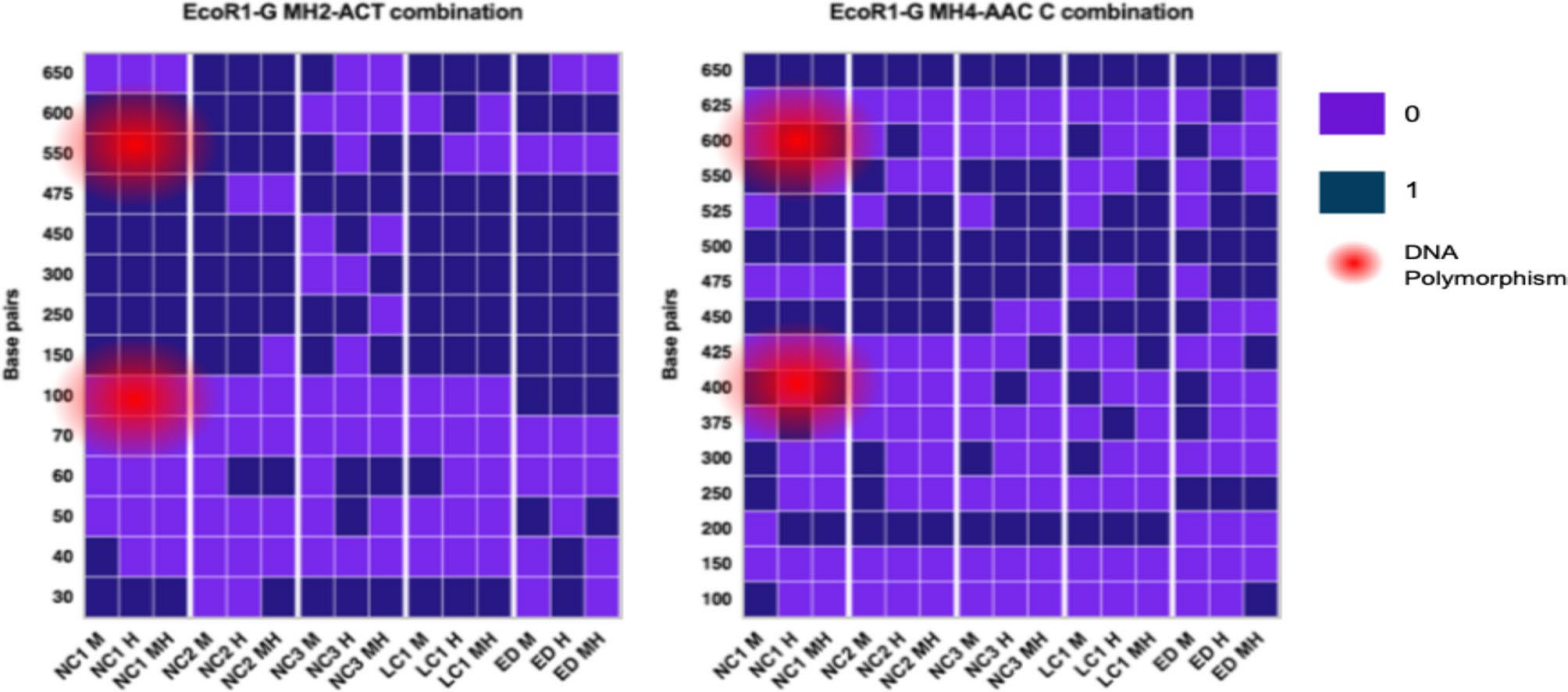
Heatmaps represents the example of methylation sensitive amplification polymorphism (MSAP) profiles in DNA methylation patterns observed in NC1 (node culture-derived shoots from liquid medium), NC2 (node culture-derived shoots from semi-solid medium), NC3 (node culture-derived plants from greenhouse), LC1 (leaf culture-derived plants from greenhouse) and ED (cutting propagated plants from greenhouse) of lingonberry cultivar Erntedank, obtained by using the primer combination *EcoR1*-G/MH2-ACT and *EcoR1*-G/MH4-AAC C. The GraphPad Prism (version 8.0.0) was used to generate the heatmap. M, H, and MH refer to the digestion with combinations of *EcoR1* + *MspI* (M), *EcoR1* + *HpaII* (H), and *EcoR1* + *MspI* + *HpaII* (MH), respectively. “0” refers to the absence of methylated DNA band, and “1” refers to the presence of methylated DNA band. In NC1, DNA bands (marked by red spot) present in M digestion lanes but not in H lanes indicate internal cytosine methylation in *EcoR1*-G/MH4-AAC C combination. NC1 banding pattern (marked by red spot) absent in M lane in *EcoR1*-G/MH2-ACT combination indicates DNA methylation polymorphisms. Likely, in NC1 banding pattern (marked by red spot) present in H digestion lane but not present in M lanes indicates external cytosine methylation in *EcoR1*-G/MH4-AAC C combination. On the other hand, in NC1 banding pattern (marked by red spot) present in both M and H lane of *EcoR1*-G/MH2-AAT combination indicates non-methylation. Ladder: 50 bp (New England Biolabs Ltd., Whitby, ON).

For the detection of methylation-sensitive DNA bands, 12 combinations of selective primers (*EcoR1* and *EcoR2*, *MspI*, and *HpaII*) were used. The methylation-sensitive DNA bands were observed in polyacrylamide gel electrophoresis (PAGE). Fully-methylated (Fmet), hemi-methylated (Hmet), and non-methylated (Nmet) sets of bands were identified at 5'-CCGG-3' sites in micropropagated shoots (NC1, NC2) and plants (NC3, LC1) and their cutting propagated donor plants (ED). In micropropagated lingonberry shoots/plants, the DNA bands were present in both lanes [*EcoR1* + *MspI* (M) and *EcoR1* + *MspI* + *HpaII* (MH)] but absent in [*EcoR1* + *HpaII* (H)], which indicates that the internal cytosine was fully-methylated [5'-C^m^CGG-3'] (Fig. 1). Similarly, the existence of methylated bands of DNA in both lanes [*EcoR1* + *HpaII* (H) and *EcoR1* + *MspI* + *HpaII* (MH)] and their absence in [*EcoR1* + *MspI*, (M)] demonstrated that the external cytosine was hemi-methylated [5'-^m^CCGG-3'], mostly observed in micropropagated lingonberry (Fig. 1). Some DNA bands were visualized in three lanes [*EcoR1* + *MspI* (M), *EcoR1* + *HpaII* (H) and *EcoR1* + *MspI* + *HpaII* (MH)] that represents non-methylation in lingonberry, mostly observed in ED plants (Fig. 1). The total number of methylated and non-methylated bands was observed in NC1 = 139, NC2 = 144, NC3 = 148, LC1 = 162, and ED = 136 (supplementary Table S2). In all lingonberry samples, the rate of methylation was LC1 > NC3 > NC2 > NC1 > ED, with LC1 producing the highest level of methylation and ED, the lowest. In this study, LC1 obtained 46 fully methylated [5'-CmCGG-3'] sites from twelve different combinations of selective primers, while scoring 62 hemi-methylated (5'-mCCGG-3') sites from the same twelve different combinations of selective primers. A PAGE heatmap visualizes the banding patterns of DNA and DNA polymorphisms in in vitro-grown shoots, and micropropagated and cutting propagated greenhouse-grown plants (Fig. 2). In this study, the primer combination *EcoR1*-G/MH2-ACT and *EcoR1*-G/MH4-AAC C were used to observe DNA polymorphism through a heatmap. M, H, and MH refers to the digestion with combinations of *EcoR1* + *Msp*I (M), *EcoR1* + *HpaII* (H), and *EcoR1* + *MspI* + *HpaII* (MH), respectively. In NC1, DNA bands were marked by a red spot, present in M and MH digestion lanes but not present in H lanes, which indicate internal cytosine methylation (Fmet) in *EcoR1*-G/MH4-AAC C combination. Whereas, in NC1 methylated DNA was marked by a red spot that absent or present in M, H, and MH lane altogether found in *EcoR1*-G/MH2-ACT primer combination.

### Analysis of secondary metabolites and their comparative study

The total phenolic content (TPC) of lingonberry node and leaf culture derived shoots and plants, and cutting propagated plants were dependent on various cofactors like environment and growing conditions. This experiment of lingonberry was notably varied by one-way ANOVA (*P* ≤ 0.05). The greenhouse plants exhibited high phenolic activity compared to the in vitro-grown node culture derived explants which might be effect of DNA methylation due to propagation methods and environmental effects under in vitro and *ex vitro* conditions. All greenhouse-grown plants had higher TPC than in vitro-grown shoots. In this study, TPC of NC3 [7.585 ± 0.0 mg gallic acid equivalents per gram of fresh leaf weight (GAE/flw)], LC1 (7.584 ± 0.0004 mg GAE/flw) and ED plants (7.584 ± 0.0004 mg GAE/flw) appeared the same. On the other hand, NC2 represented lowest TPC (2.483 ± 0.982 mg GAE/flw) and it was followed by NC1 (3.791 ± 0.732 mg GAE/flw).

Different growing conditions had also effects on total flavonoid content (TFC). One-way analysis of variance (ANOVA) showed significant differences among the treatments (P ≤ 0.05). The TFC was highest in ED plants and lowest in NC2. The decreasing order of TFC was NC2 [3.264 ± 1.138 mg catechin equivalents per gram of fresh leaf weight (CE/flw)], < LC1 (4.490 ± 0.303 mg CE/flw) < NC1 (6.240 ± 0.422 mg CE/flw) < NC3 (7.260 ± 1.575 mg CE/flw) < ED (7.917 ± 0.384 mg CE/flw).

Total antioxidant activity/capacity (TAC) of micropropagated and cutting propagated shoots/plants were analyzed using DPPH assay and the treatments varied significantly as was observed by one-way ANOVA (P ≤ 0.05). The decreasing order of total antioxidant activity was ED (0.0350 ± 0.0014 mg GAE/flw) ≤ NC1 (0.0350 ± 0.0012 mg GAE/flw) < LC1 (0.0353 ± 0.0008 mg GAE/flw) < NC3 (0.0356 ± 0.0014 mg GAE/flw) < NC2 (mg GAE/flw).

In vitro propagated shoots and *ex vitro* propagated plants were significantly different for total proanthocyanidin content (TPrC; ANOVA, P ≤ 0.05) which might be the DNA methylation effects due to propagation method and culture environment under in vitro and *ex vitro* conditions. The TPrC was highest in ED and lowest in LC1 with the decreasing order of LC1(0.0013 ± 0.0003 mg CE/flw) < NC2 (0.0018 ± 0.0004 mg CE/flw) < NC3 (0.0024 ± 0.0013 mg CE/flw) < NC1 (0.0033 ± 0.0007 mg CE/flw) < ED (mg CE/flw).

### Correlations of secondary metabolites

**Figure 3:**
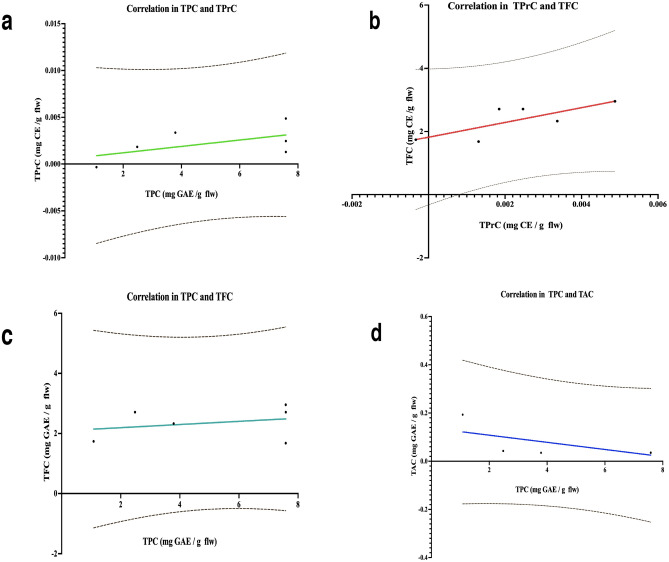
Linear regression in secondary metabolites of lingonberry shoots in vitro and plants under *ex vitro* condition. Data were analyzed based on means ± SD, *n* = 6. Significant differences not present at *α* = 0.05 by Spearman test. (**a**) Correlation between TPC and TPrC. (**b**) Correlation between TPrC and TFC. (**c**) Correlation between TPC and TFC. (**d**) Correlation between TPC and TAC.

All secondary metabolites (TPC, TPrC, TAC, TFC) of in vitro and *ex vitro*-grown shoots/plants of lingonberry cultivar Erntedank had a significant relationship. These data were analyzed by linear regression in (GraphPad Prism 8.0.0 software). The TPA was directly proportional to TPC (Fig. 3a), TPrC to TFC (Fig. 3b) and TPC to TFC (Fig. 3c), while TPC was inversely proportional to TAC (Fig. 3d).

### Portraying the partnerships of cytosine methylation and secondary metabolites

**Figure 4:**
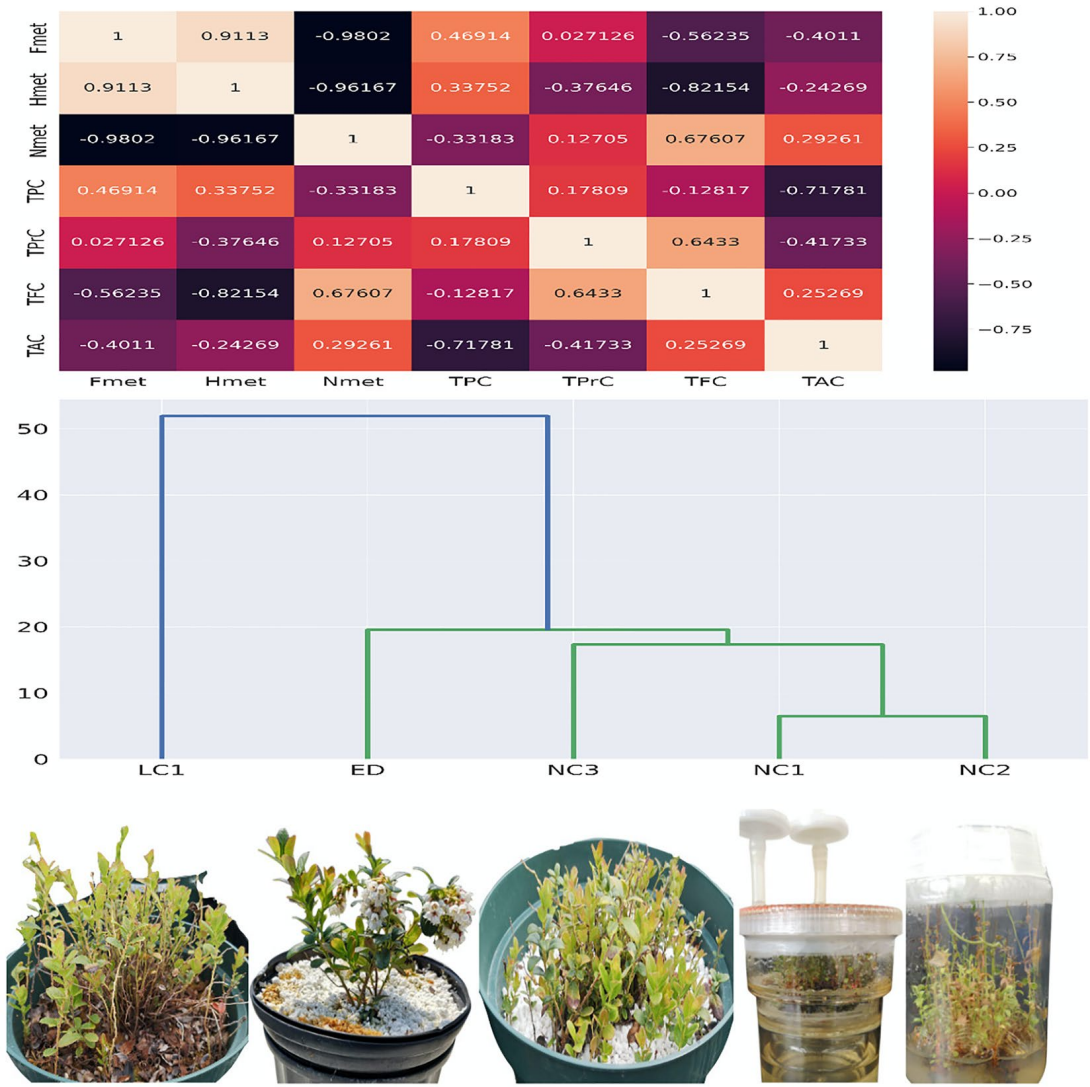
Heatmap represents the partnership between cytosine methylation and secondary metabolites in lingonberry. Dendrogram portraying that node culture-derived in vitro propagated shoots NC1 and NC2 were closely related to node culture-derived greenhouse-grown plant NC3 than those of LC1 and ED. The matplotlib package in Python was used to generate the heatmap. Fmet refers to full-methylation, Hmet refers to hemi-methylation and Nmet refers to non-methylation. TPC = total phenolic content, TFC = total flavonoid content, TPrC = total proanthocyanidin content and TAC = total antioxidant activity. NC1, NC2, NC3 and LC1 are the micropropagated shoots and plants, and ED is cutting propagated plants of lingonberry cultivar Erntedank (more details are in supplementary Table S3).

DNA methylation plays a critical role in the regulation of secondary metabolites. Heatmap (Fig. 4) demonstrate the Pearson correlation between cytosine methylation and secondary metabolites (TPC, TPrC, TFC, TAC). Data were analyzed by matplotlib. There is a positive correlation among Fmet, TPC, and TPrC but TFC and TAC showed a negative correlation. Conversely, TPrC is slightly positive and TAC is slightly negative, but TFC represents strongly negative relationship. Hmet has only one positive correlation with TPC. Among all negative relations (TPrC, TFC, TAC); TFC displayed the highest level of negative correlation with Hmet. When secondary metabolites were compared, Nmet and Hmet had an inverse relationship. Among all of the secondary metabolites, TPC showed negative relation with Nmet, but TFC showed a strong positive relationship.

In this study, the in vitro-grown lingonberry shoots NC1 and NC2 showed a higher methylation rate but had comparatively low secondary metabolites (Supplementary Table S3). NC2 had the lowest TPC (Fig. 4). Consequently, LC1 had a low Nmet but a higher rate of methylation occurrence and more TPC content. NC3 displayed high Nmet but had an equal amount of TPC like LC1 (Fig. 4). TFC was high in NC1 and NC2, where a higher methylation rate appeared. However, TFC was low in LC1 and high in ED. LC1 showed the lowest TPrC where methylation rate is the highest but Nmet was the lowest. Finally, NC3 and NC1 appeared to have low level of TAC; on the other hand, LC1, NC2, and ED had a same amount of TAC. From the dendrogram, we observed that NC1, NC2, and NC3 were closely related than LC1 and ED (Fig. 4). The study demonstrated that the total number of methylated bands is inversely related to all secondary metabolites in different propagated methods of lingonberry.

## Discussion

Epigenetic variation influences complex traits in plants. In epigenetic insight, genetic sequences remain the same during different developmental stages but the plants are phenotypically different^31^. Many genes have been epigenetically modified through cytosine methylation, as demonstrated through the MSAP assay^32^. As a result, gene expression has been altered by DNA methylation modification. Genetic and epigenetic variations occur in tissue culture plants due to environmental stress^33^. MET1 is the leading cause of methylation, which was present in the regenerated plants^34^. Our investigation showed that the growth regulator zeatin (used in in vitro shoot proliferation medium) and indole-3-butyric acid (IBA; used for rooting of microshoots) affected in vitro-derived plants, indicating both hyper- and hypo-methylation. While hyper-methylation inhibits the expression of ARF3 gene (Auxin Response Factor-3), resulting in apical dominance of in vitro-derived plants; hypo-methylation increases ARF3 gene expression^35,36^. In the present study, the vibrant variations of DNA methylation during in vitro and *ex vitro* culture of lingonberry cultivar Erntedank could be due to many factors including culture environment, media type, and types and concentration of plant growth regulators used in culture media. The higher DNA methylation was monitored in suspension cultures containing cytokinin than those of auxin in eggplant^37^. Due to a higher concentration of thidiazuron, the methylation rate has been reduced in blueberry callus^38^. Our results agree with the blueberry cultivar Fundy that expressed higher DNA methylation in micropropagated plants than those of tissue culture ones^36^. In blueberry, Ghosh et al.^38^ reported increased methylation percentage in in vitro-grown shoots in a liquid medium compared to those on a semi-solid medium. In the current study, Erntedank cutting propagated plants were used as control; thus the cytosine methylation was observed in node culture-derived shoots in a liquid and on a semi-solid medium, along with node and leaf culture-derived plants in a greenhouse where highest methylation was expressed in leaf culture-derived plants (Figs.1, 2).

The secondary metabolites TPC, TFC, TAC, and TPrC were synergistically and antagonistically affected by various environmental factors and ages of lingonberry shoots and plants. Significant interactions among all propagated plant in different growth conditions was reported where TPC, TFC, TAC, and TPrC, were higher in tissue culture plants than cutting propagated ones in lowbush blueberry^39^. TPC, TFC, and TPrC contents were higher in greenhouse-grown cutting propagated ED plants than in tissue culture shoots and plants which might be the effects of DNA methylation due to propagation method and culture environment under in vitro and *ex vitro* conditions. The ED plants had low antioxidant activity. Other study in lingonberry reported that TFC was highly correlated with TAC^7^. We analyzed using linear regression and predicted a relationship between secondary metabolites and each propagated plants based on our data. TPC was directly proportional to TPrC, exhibiting a positive relationship. TPrC correlated to TFC was found in our study. TPC was proportional to TFC but inversely proportional to TAC. While TAC decreased in in vitro and *ex vitro*-grown shoots/plants, TPC increased (Fig. 3). High correlation among TPC, TPrC, and TAC was also reported in in vitro and *ex vitro* propagated blueberries^39^.

Variation in DNA methylation in micropropagated plants were also found in *Rhododendron* where tissue culture plants displayed 12.17% nonmethylation at 5'-CCGG-3' sites compared to donor mother plants^40^. In our study, tissue-culture LC1 showed 8.58% more cytosine methylation than maternal genotype ED, but NC1, NC2, and NC3 had low methylation rates than LC1 (4.85%, 6.7, and 12.14%, respectively). Among 24,794 bands in PAGE, 26.61% methylated bands were observed in *Rhododendron*^40^. It has been reported that DNA methylation increased in floral buds while decreased in vegetative buds^41^. In blueberry, methylation rate increased in micropropagated plants compared to conventional cutting propagated plants^36,38^. Similarly, thidiazuron-induced blueberry calli exhibited an increased methylation rate compared to cutting plants^38^. In the present study, we compared each micropropagated plants with a cultivar using the MSAP assay, and the methylation rate increased from in vitro regenerates to acclimatized tissue-cultured greenhouse propagated plant. In leaf regenerants (LC1), we obtained 14 more methylation bands than node culture-derived regenerants (NC3), where the fully-methylated and hemi-methylated rate was the highest among all propagated plants. Therefore, we can summarize the total methylated segments present in each clone where the most elevated amount was exhibited by LC1 (162 bands) and lowest present by ED (136 bands) (Figs.1 and 2; supplementary Table S2). The fully-methylation level was high, and the hemi-methylated level was low for the lingonberry genome, which is compatible with previous studies in blueberries^36^ and *Agave tequilana*^42^.

Due to several environmental factors including stresses during in vitro culture^15,23^, DNA methylation regulates gene expression. Altered DNA methylation leads to improved plant disease resistance and drought stress tolerance by recruiting chromatin remodelers histone deacetylases and histone methyltransferases to repress transcription^43^. In plants, inhibited DNA methylation could increase or decrease secondary compounds, observed in *Taxus* spp., *Salvia miltiorrhiza*, and *Vitis amurensis*^44,45^. Our report exhibited the inverse correlation between DNA methylation with secondary metabolites (Fig. 4). Firstly, TPC had strong positive relationship with Fmet and Hmet which reflected that TPC is directly proportional to cytosine methylation. Secondly, TPrC had slightly positive relations with Fmet and Hmet which means TPrC is slightly proportional to cytosine methylation. Thirdly, TFC was highly inversely proportional to cytosine methylation. Finally, TAC was slightly inversely proportional to cytosine methylation. The cytosine analog 5-azacytosine dramatically increased phenolic acid accumulation and expressions of key genes involved in the phenolic acid biosynthesis pathway. However, decreased methylation levels of CG and CHG sites were found. CHH methylation helps in the synthesis of the rosmarinic acid synthase gene (RAS) as a promoter^29^.

Similarly, we observed flavonoids and proanthocyanidin also represented an inverse relationship with cytosine methylation. On the other hand, the formation of 'double lock' cooperation was observed between DNA methylation and histone modification^46–48^. Previous research found higher cytosine methylation occurred with high levels of native secondary metabolites in the autopolyploid *Cymbopogon sprengel*^49^. We speculated the same trend in antioxidant activity, where 50% methylation was inversely proportional to total antioxidant activity among all environmental factors. Fully-methylated DNA compared with TPC, we found that both *ex vitro* and in vitro specimen exhibited high methylation with low phenolic content. Enzymes like PAL (phenylalanine ammonia lyase), 4CL (4-coumarate-CoA ligase), C4H (cinnamic acid 4-hydroxylase), TAT (tyrosine aminotransferase), HPPR (4-hydroxyphenylpyruvate reductase), CYP98A14, and RAS (rosmarinic acid synthase) were identified for phenolic acid biosynthesis by DNA methylation in *S. miltiorrhiza* hairy roots^45^. Increased DNA methylation reduces the expression of PAL and CYP98A14 at the level of 16.7% and 45.5%, respectively. However, decreased methylation enhances RAS expression^45^. The expression of the VaSTS10 gene was significantly increased with a decrease in methylation^49^. This study depicted that cytosine methylation has a converse relationship with secondary metabolites of lingonberry among all types of propagated shoots and plants. Current study suggests the existence of a dynamically changing cytosine methylation during the in vitro culture of lingonberries that might play a significant role in controlling gene regulation and expression at different propagation stages, leading to the production of various secondary metabolites.

## Conclusion

In the present study, micropropagation enhances the rate of secondary metabolite concentration in lingonberry. However, those effects were genotype-specific. Overall, leaf culture regenerated plant in the greenhouse showed better phytochemical content. This study proved that in vitro propagated greenhouse plants had tissue-specific effects from phytochemical characteristics and phenotypic expression in lingonberry.

A large body of data library in lingonberry epigenetic study will be used as new efficient tools for understanding the origin of lingonberry, evolution, and taxonomy. The data will help to characterize various types of epigenomic changes for epimutation. They will help in identifying the potential tissue culture-derived health promoting lingonberry plants. This is undoubtedly to say that DNA methylation will serve as the important biotechnological tool to cover our current increasing food demand in the sense of quality and quantity of commercial lingonberry production.

## Materials and methods

### Plant material and shoot proliferation in vitro on a semi-solid medium and in a bioreactor containing liquid medium

In vitro-grown shoots and greenhouse-grown tissue culture and cutting propagated plants of lingonberry cultivar Erntedank^50^ were used for this study and the study was conducted at St. John’s Research and Development Centre (SJRDC), Agriculture and Agri-Food Canada, Newfoundland and Labrador, Canada. The plants used in the experiment comply with proper institutional, national, and international guidelines and legislation. The cultivar Erntedank and the leaf culture-derived plants of cultivar Erntedank developed at SJRDC^50^ were maintained in a greenhouse in plastic pots containing two parts peats and one-part perlite (v/v) and maintained in a greenhouse at 20 ± 2 °C, 85% relative humidity, and 16-h photoperiod at a maximum PPF of 90 μmol m^−2^ s^-1^ under the supervision of Dr. Samir C. Debnath (the corresponding author). Dr. Debnath has been conducting research with these plant materials at SJRDC^50^ and undertook the formal identification of the plant material used in this study. Node culture-derived shoots were established in vitro following the protocol of Debnath^50^. Shoots proliferated from nodal explants were divided into three-node stem sections and cultured on a semi-solid medium in 175- mL jars (Sigma Chemical co., St. Louis, U.S.A.) containing 35 ml nutrient medium D^51^, including 25 g L^−1^ sucrose, 3.5 g L^−1^ agar, 1.25 g L^−1^ Gelrite (Sigma Chemical Co.) and 1 µM zeatin (NC2). Another culture (NC1) was set in Growtek stationary bioreactors (Growtek^tm^ culture vessels, Fischer Scientific, Ottawa, Ontario, Canada) using 200 mL of the same medium but without agar and Gelrite (liquid medium)^10^. The experiment was replicated three times. There were five explants on a semi-solid medium and eight in the liquid medium. Proliferated shoots were sub-cultured every 8-weeks in a fresh medium^10^.

### Evaluation of tissue culture derived and cutting propagated plants under greenhouse condition

Node (NC3) and leaf culture-derived shoots (LC1) along with terminal cuttings of lingonberry cultivar Erntedank were established in a greenhouse following the protocol of Debnath^52^. Briefly, node and leaf culture-derived tissue culture shoots and stem cuttings of cultivar Erntedank were treated with IBA (39.4 mM) and transferred to 45 cell plug trays containing peat-perlite (2:1, v/v) medium and maintained in 95% humidity at 22 ± 2 °C, 16-h photoperiod 55 µmol m^−2^ s^−1^ for rooting. Leaf culture-derived tissue culture plants were obtained through adventitious shoot regeneration followed by rooting of microshoots and acclimatization in a greenhouse^50^. After six weeks, the surviving plants were transferred to a greenhouse and grown following a previous study protocol^52^. Cutting propagated and tissue culture plants were maintained for more than ten years. There were 5 plants in each treatment and the experiment was replicated five times.

### Data collection

Morphological data were collected from three randomly selected explants from liquid and semi-solid media, replicated three times for in vitro-grown shoot cultures after eight weeks of culture, and three randomly selected plants in two-year-old greenhouse-grown plants in August 2018 when the plants are in full growth. While in vitro-cultured shoots were grown at 20 ± 2 °C with 16 h photoperiod of 30 μmol m^−2^ s^−1^ photosynthetic photon flux (PPF) light intensity, greenhouse-grown plants were maintained with 90 μmol m^−2^ s^–1^ PPF maximum light intensity at 20 ± 2 °C, 85% humidity^10^. The morphological data of in vitro-grown shoots, and of micropropagated and conventionally propagated plants in greenhouse were collected^10^. Shoot vigour and plant vigor were determined by visual assessment, ranging from scale 1 (very poor) to 8 (fully normal and healthy plants with large green leaves and excellent vigor). Shoot and leaf characteristics were recorded from three fully expanded growing mature shoots selected randomly for both liquid and semi-solid media^10^.

### DNA isolation

For both DNA and biochemical components analyses, young leaves were plucked from eight-week-old in vitro-grown shoots and from two-year-old greenhouse-grown plants in August, 2019, and immediately frozen in liquid nitrogen and stored at − 80 °C. Genomic DNA was isolated from 100 to 145 mg of young lingonberry leaves. DNeasy Plant Mini Kits (Qiagen GMbH, Hilden, Germany) was used and followed the manufacturer's instructions. DNA concentration ranges from 55 to 150 ng µL^−1^, and the absorbance ratios A260/A280 and A260/A230 of 1.8–1.9 and 2.1–2.4 respectively.

### Methylation-sensitive amplification polymorphism (MSAP) assay

MSAP assay is the modified version of the AFLP protocol^38^. This assay was performed the experiment three times to detect MSAP digestions; methylation-sensitive restriction enzymes (isoschizomers) *EcoRI*, *MspI*, and *HpaII* (Thermo Scientific) were used in this study. Isolated DNA samples were digested for 1.5 h at 37 °C with the restriction enzyme of 75 µL *EcoRI* (#FD0274, Thermo Fisher Scientific, Waltham, MA) and then, 15-min incubation at 65 °C where *EcoRI* enzyme was activated. Then digested DNA was separated into three parts: *MspI*, *HpaII*, and *MspI* + *HpaII*. After that, the total volume containing 10X Fast Digest buffer was incubated for 3 h at 37 °C and then 15 min at 65 °C, where digestion was carried out. The digested DNA was ligated with a combination of EcoRI adapter, MspI and *HpaII* adapter in a 100 µL reaction containing ligase buffer, T4 DNA ligase (#EL0014, Thermo Fisher Scientific), and 50% of polyethylene glycol solution. The ligation was done for 5 h at 23 °C, and then 10 min at 65 °C to stop the ligation. Ligated fragments were pre-amplified using pre-selective complementary primers (Table S1). Pre-amplified products were assessed by 1.8% agarose gel electrophoresis, where visible smear was observed from 100 to 1000 bp. Pre-amplified products were diluted five times with 0.1X T.E. buffer. Diluted pre-amplified products were performed in selective amplifications with a combination of selective primers. After that, the total number of selective primers and their twelve combinations were used (Supplementary Table S1). Selective amplifications were carried out with the combinations of two EcoRI forward primers (EcoRI 1 and EcoRI 2) and six MspI- *HpaII* reverse primers (MH1 to MH6), selective amplification was assembled using master mix 1X PCR buffer in 25 µL final volume. Selective-amplified products were visualized using 6% denaturing polyacrylamide gel electrophoresis (PAGE). The gel was run at 55 V for 3 h and 35 min. The DNA fragments were stained with PAGE GelRed™ dye and visualized to detect the molecular-sized marker compared to a 50 bp ladder. The DNA fragments showed reproducible results between replicates. Each sample has been divided into three digested samples through *MspI/HpaII* adapters. With the help of 12 selective primers, 3 pre-amplified samples turned into 36 samples and thus, 5 samples turned into 180 samples. More details are in supplementary information (Supplementary information Fig. S2)^53^.

### Leaf extraction for secondary metabolites

100 mg of fresh young leaves were collected from the greenhouse and growth chamber, and stored at − 80 °C in liquid nitrogen. Pre-frozen extract leaves were homogenized in a homogenizer (FastPrep-24 Tissue and Cell Homogenizer M.P. Biomedicals, Irvine, CA, U.S.A.) containing 80% aqueous acetone solution and 0.2% formic acid (1:4 g/mL). Subsequently, the homogenate was kept as slow agitation at 4 °C for 30 min and then centrifuged at 13,000 rpm in 15 min at 4 °C (Allegra 64R Beckman Coulter Inc., Palo Alto, CA, U.S.A.). The final volume of the secondary metabolic crude extract was preserved in the ultralow freezer (Thermo Scientific, Burlington, ON, Canada). For further chemical analysis, three replication were used.

### Estimation of the total phenolic content

Total phenolic contents were measured using Folin-Ciocalteu reagent, an acidic phosphomolybdotungstate solution where oxidized phenolates blue color were formed ^39^. Diluted extract samples were treated with 100 mL of Folin-Ciocalteu reagent and 200 mL of saturated sodium carbonate and then mixed gently by adding 1.5 mL distilled water. The reading of absorbance was taken at 725 nm against the blank. Total phenolic content (TPC) was detected by gallic acid equivalents (GAE) mg/g flw (fresh leaf weight). In our study, we used gallic acid equivalents as a standard^54^.

### Estimation of the total flavonoid content

The flavonoid content of lingonberry samples was analyzed by the colorimetric method^55^. Extracted samples and standard solution of catechin were added with 2 mL of distilled water, 150 mL of 5% (w/v) sodium nitrite and 150 µl of 10% (w/v) aluminum chloride. It was measured at 510 nm against the blank. The total flavonoid content (TFC) of leaves was expressed with as catechin equivalent (CE) as standard, and the unit is mg/g flw^56^.

### Estimation of the total antioxidant activity

2,2-diphenyl-1-picrylhydrazyl (DPPH) having the scavenging effect was performed for the estimation of antioxidant activity of leaf extracts, and gallic acid equivalent (GAE) was used as a standard for the expression of the total antioxidant assay^6,38^. 100 mL of diluted extract solution and the standard solution was mixed gently with 1.7 mL of methanol, 0.06 mM DPPH solution, and 80% aqueous acetone as blank. Extracted leaf samples, standard GAE and blank solutions were incubated at room temperature and kept in the dark for 45 min; the absorbance was measured at 517 nm. The scavenging activity was derived from the following formula:

DPPH scavenging % = [(A_517nm(Blank)_ − A_517nm(Extract)_)/A_517nm(Blank)_] × 100, where A = absorbance.

### Estimation of the total proanthocyanidin content

Leaf extract was investigated for determining proanthocyanidin content using the modified vanillin technique^57^. 0.5 mL of diluted extracts and standard catechin equivalents (CE) was added in 0.5% vanillin-HCL reagent (2.5 mL). Then, the solutions were mixed and incubated in the dark for 20 min. Catechin has a range of 50 to 500 µm concentration, which is the standard for proanthocyanidin expression^56^. Thus, the absorbance was measured at 500 nm. Total proanthocyanidin (TPrC) content was denoted by CE mg/g flw.

### Statistical analysis

In the current studies, the morphological data and MSAP assay were analyzed by using GraphPad Prism 8.0.0 software^58^. For statistical analysis, t and Wilcoxon test were evaluated at *α* = 0.05 for all the parameters^59^. All morphological data are expressed as the means ± SD of three replications. The treatment means were compared by the least significant difference (LSD) using the t and Wilcoxon test. Data of secondary metabolites was performed by one way ANOVA with a standard significance threshold of *p* < 0.05. Correlation of secondary metabolites (TPC, TFC, TPrC,TAC) were performed through linear regression by Spearman test. DNA methylation events and secondary metabolites were correlated by Python matplotlib^60^.

## Supplementary Information

Supplementary Information.
