# Supplementary material for: Epigenomic insight of lingonberry and health-promoting traits during micropropagation
Source: Sci Rep. 2022 Jul 21;12:12487. doi: 10.1038/s41598-022-16530-7 (PMC9304418; doi:10.1038/s41598-022-16530-7)

Supplementary information

Figure S1. Global distribution of lingonberry in different parts of the world. The map was generated using MS-excel (MS-365).

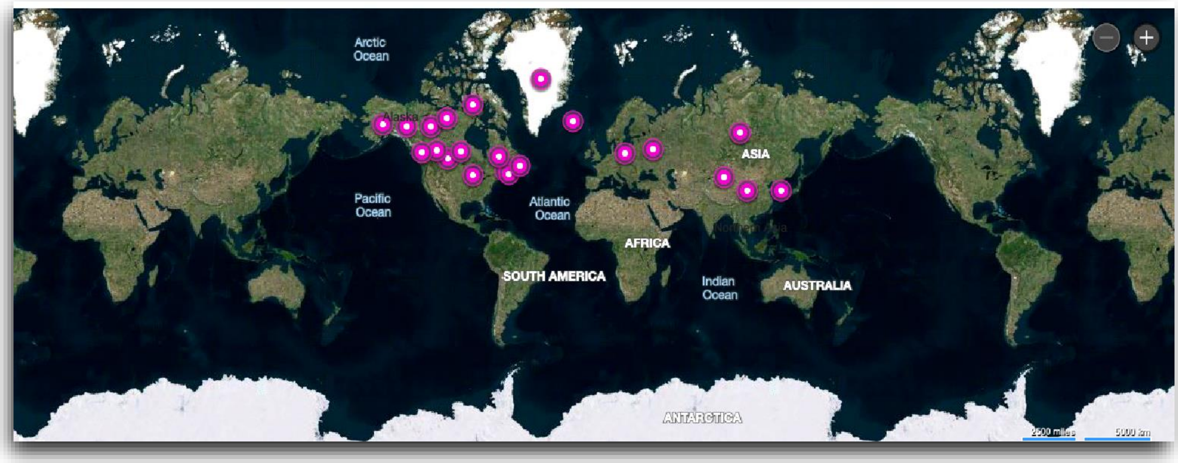

Figure S2. Mechanism of DNA methylation using Methylation Sensitive Amplification Polymorphism (MSAP) assay. The diagram was generated using mind the graph software (https://mindthegraph.com/).

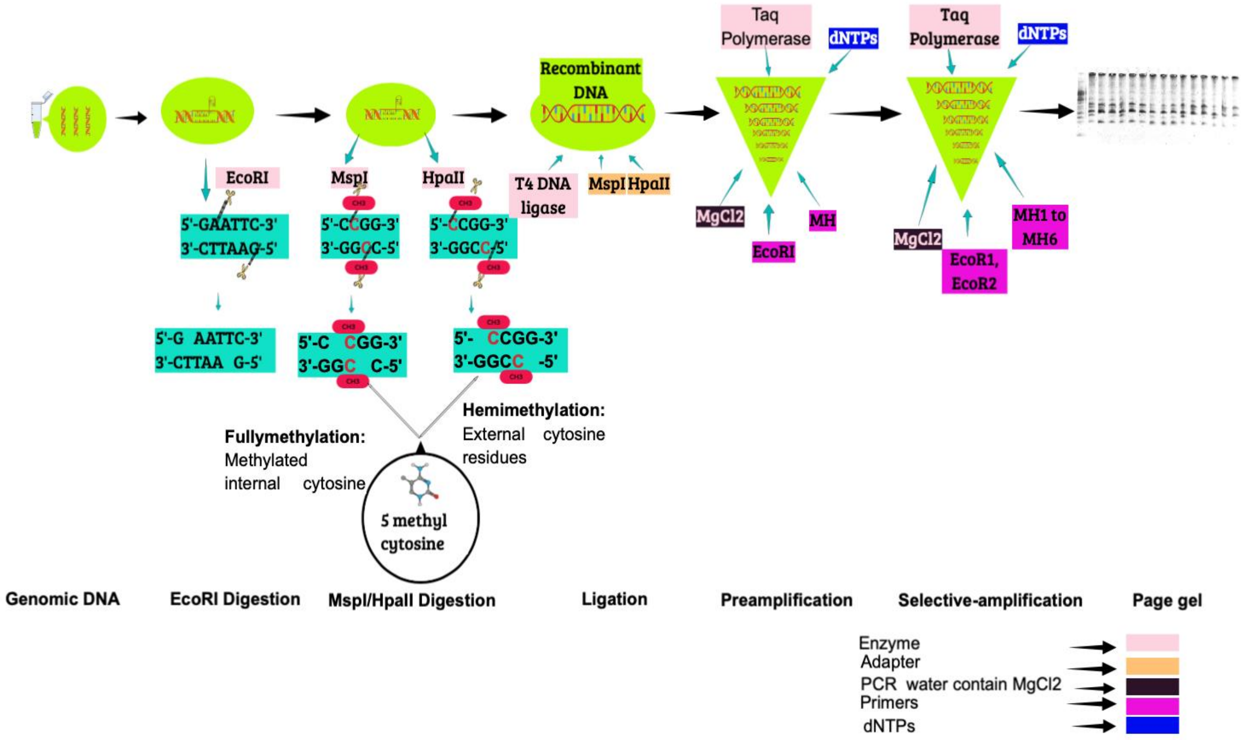

Table S1. Sequences of adapters and primers performed in MSAP assay.

**Adapters:**	
EcoRI:	5'- CT GTA GAC TGC GTA CC -3'
	3'- CA TCT GAC GCA TGG TTAA -5'
MspI-HpaII:	5'- GA TCA TGA GTC CTG CT -3'
	3'- AG TAC TCA GGA CGA GC -5'
**Pre-selective primers:**	
EcoRI:	5'- GAC TGC GTA CCA ATT CA -3'
MspI-HpaII (MH):	5'- ATC ATG AGT CCT GCT CGG -3'
**Selective primers:**	
EcoRI 1:	5'- GAC TGC GTA CCA ATT CAC G -3'
EcoRI 2:	5'- GAC TGC GTA CCA ATT CAC T -3'
MH1:	5'- ATC ATG AGT CCT GCT CGG AAT -3'
MH2:	5'- ATC ATG AGT CCT GCT CGG ACT -3'
MH3:	5'- ATC ATG AGT CCT GCT CGG TCC -3'
MH4:	5'- ATC ATG AGT CCT GCT CGG AAC C -3'
MH5:	5'- ATC ATG AGT CCT GCT CGG CGA A -3'
MH6:	5'- ATC ATG AGT CCT GCT CGG TAG C -3'

Table S2. Summary of total number of bands, number and percentage (%) of DNA methylation events detected by methylation sensitive amplification polymorphism (MSAP) technique using twelve selective primer combinations in five samples of lingonberry.

Those samples were derived from cutting propagated (ED) greenhouse grown plants and from micropropagated shoots (NC1, in a liquid medium; NC2, in a semi-solid medium) and greenhouse grown plants (NC3, node culture-derived; LC1, leaf culture-derived).

Combinations of Selective Primers	NC1			NC2			NC3			LC1			ED		
	fmet	hmet	nmet	fmet	hmet	nmet	fmet	hmet	nmet	fmet	hmet	nmet	fmet	hmet	nmet
**Ecor1-G MH1-AAT**	3	3	6	3	2	6	2	1	7	3	3	7	3	3	8
**Ecor2-T MH1-AAT**	5	4	6	4	6	7	5	5	8	9	6	4	5	7	4
**Ecor1-G MH2-ACT**	0	1	5	2	2	4	4	4	4	1	2	4	2	2	3
**Ecor2-T MH2-ACT**	4	3	9	4	2	10	8	4	6	3	9	1	5	1	9
**Ecor1-G MH3-TCC**	3	4	7	2	4	8	3	3	7	4	4	7	3	3	4
**Ecor2-T MH3-TCC**	2	2	4	4	2	3	3	2	5	3	8	2	2	4	2
**Ecor1-G MH4-AAC C**	4	4	4	2	3	6	3	3	6	5	6	4	6	6	3
**Ecor2-T MH4-AAC C**	4	2	7	4	3	5	3	2	8	5	7	7	3	2	6
**Ecor1-G MH5-CGA A**	1	2	9	2	3	7	0	1	9	2	3	6	4	2	6
**Ecor2-T MH5-CGA A**	3	4	2	4	4	4	2	4	6	6	7	2	6	4	2
**Ecor1-G MH6-TAG C**	3	3	4	3	4	4	0	0	7	3	3	4	3	3	4
**Ecor2-T MH6-TAG C**	5	5	1	2	3	6	3	3	7	2	4	6	0	0	6
**Total bands of 3 set of methylated sites (fmet, hmet and nmet)**	37	37	65	36	38	70	36	32	80	46	62	54	42	37	57
**Total bands of in vitro-grown shoots and greenhouse-grown plant**	139			144			148			162			136		
**Total methylation (%) of in vitro-grown shoots and greenhouse-grown plant**	56.92	56.92	46.76	51.42	54.28	48.61	45.00	40.00	54.05	85.18	114.81	33.33	73.68	64.91	41.91
**Total methylated polymorphisms of in vitro-grown shoots and greenhouse-grown plant**	51			52			46			80			64		

Table S3. Data were performed for expressing the relationship between cytosine methylation and secondary metabolites. This tables were generated from matplotlib package in python.

Propagated Plants	Fmet	Hmet	Nmet	TPC	TPrC	TFC	TAC
NC 1	56.9200	56.9200	46.7600	3.7913	0.0034	2.3293	0.0350
NC 2	51.4200	54.2800	48.6100	2.4827	0.0018	2.7103	0.0427
NC 3	45.0000	40.0000	54.0500	7.5850	0.0025	2.7103	0.0357
LC 1	85.1800	114.8100	33.3300	7.5843	0.0013	1.6760	0.0353
ED	73.6800	64.9100	41.9100	7.5843	0.0049	2.9557	0.0350

All gel blots obtained from MSAP assay

Genomic DNA with 1kb, 100bp, 50bp Ladders NC1, NC2, NC3, LC1, ED performed in agarose gel electrophoresis

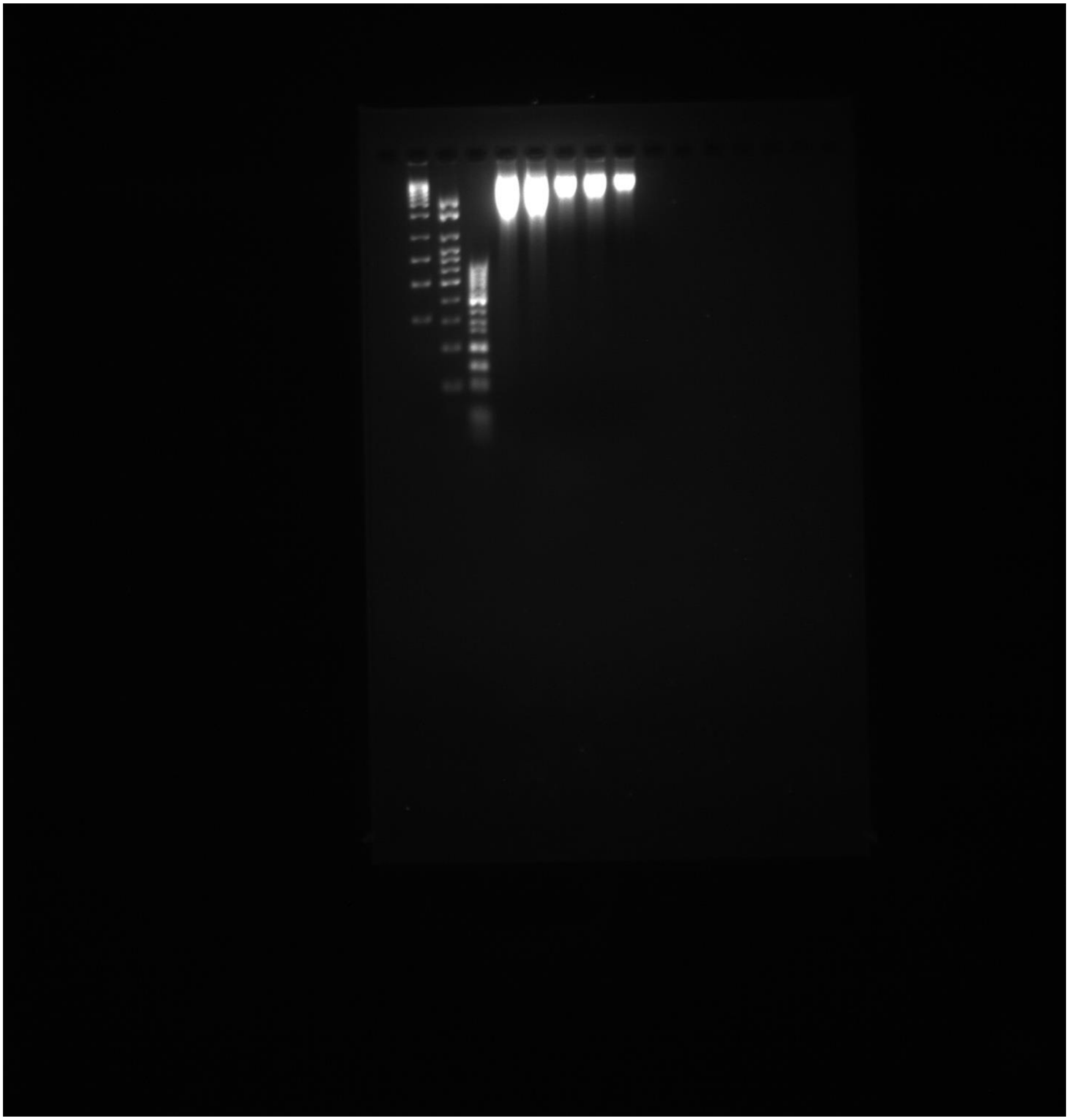

Preamplification with 100 bp NC1(MHC lanes), NC2(MHC lanes), NC3(MHC lanes), LC1(MHC lanes), ED(MHC lanes) performed in agarose gel electrophoresis

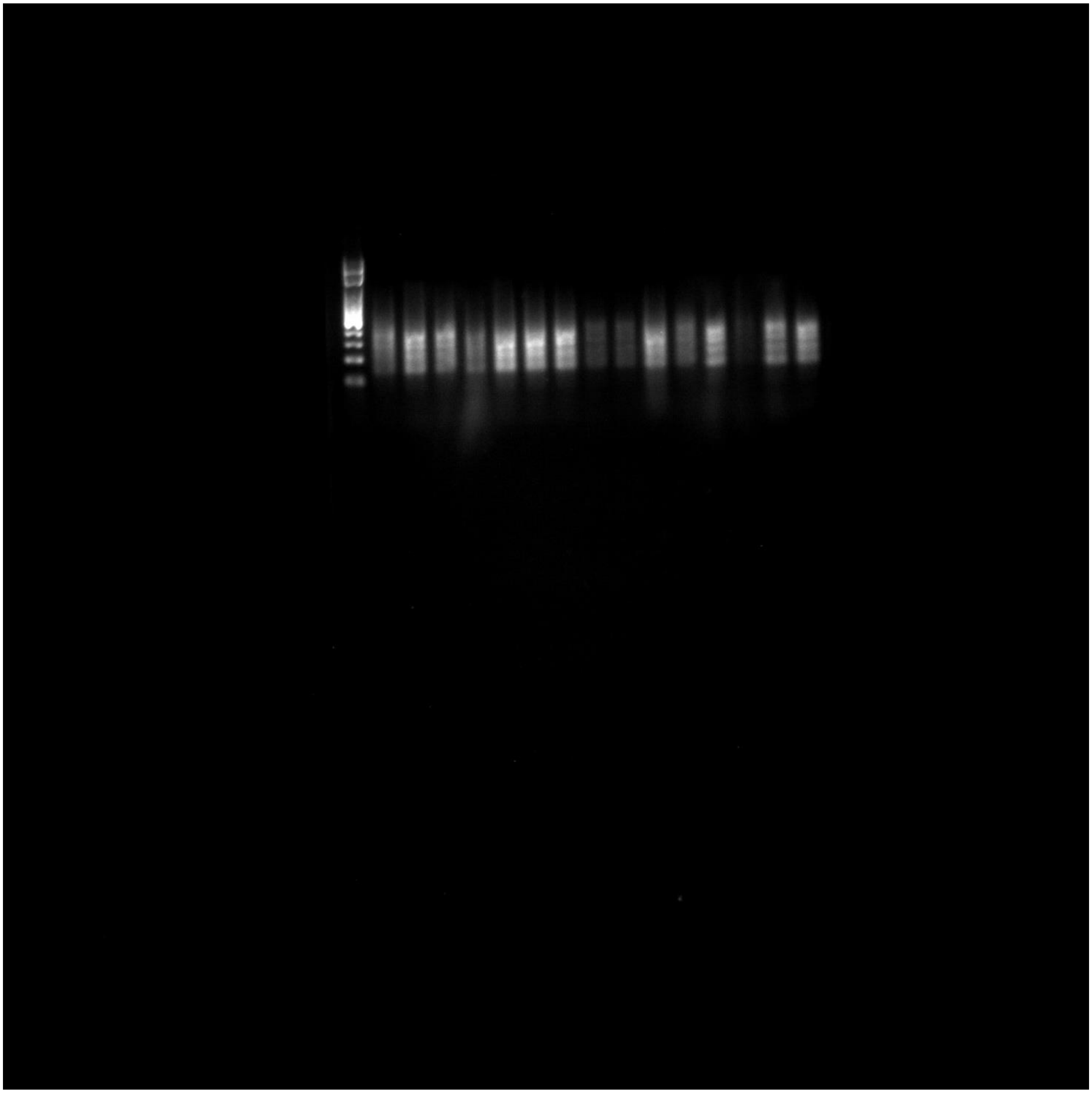

Selective amplification performed in page gel electrophoresis

EcoR1-G+MH1-AAT combination; 100 bp and 50bp NC1(MHC lanes), NC2(MHC lanes), NC3(MHC lanes), LC1(MHC lanes), ED(MHC lanes)

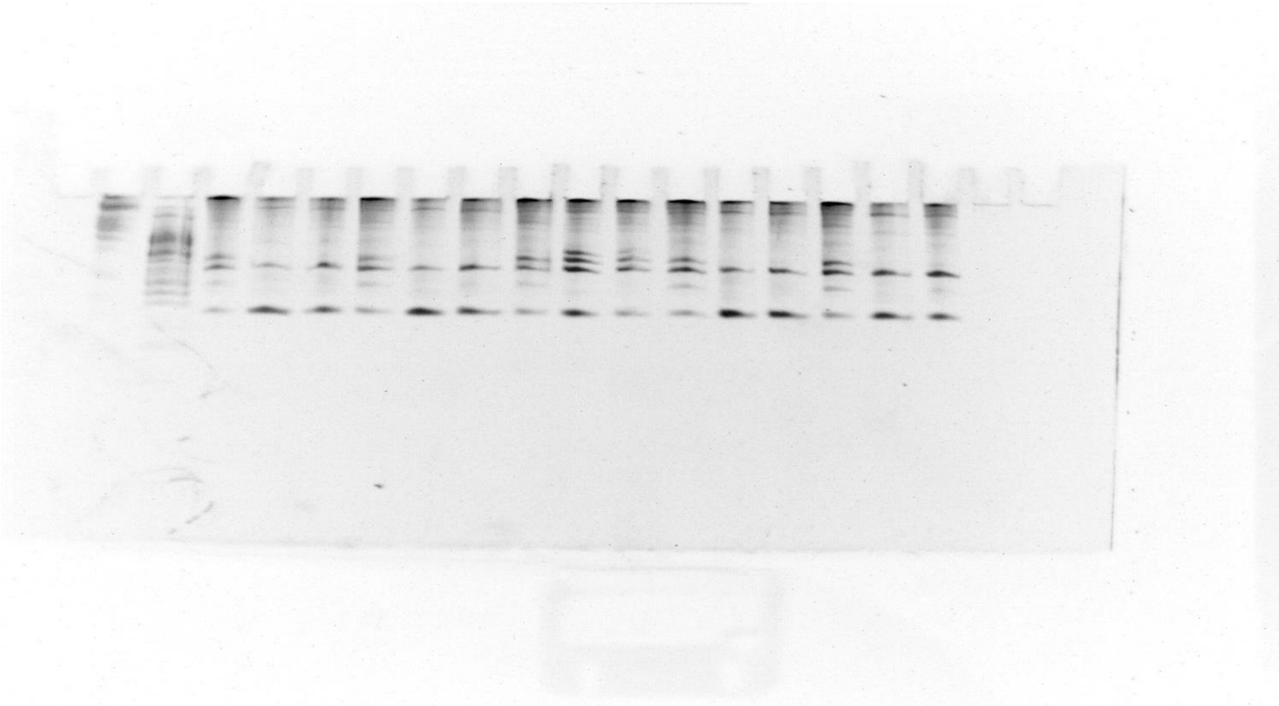

EcoR2-T+MH1-AAT combination; 100 bp and 50bp NC1(MHC lanes), NC2(MHC lanes), NC3(MHC lanes), LC1(MHC lanes), ED(MHC lanes)

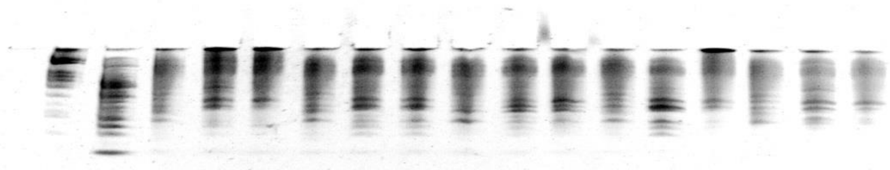

EcoR1-G+MH2-ACT combination; 100 bp and 50bp NC1(MHC lanes), NC2(MHC lanes), NC3(MHC lanes), LC1(MHC lanes), ED(MHC lanes)

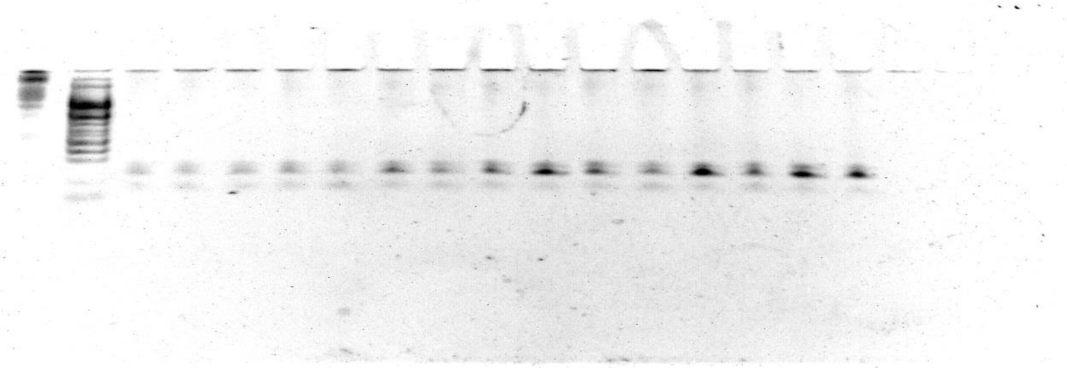

EcoR2-T+MH2-ACT combination; 100 bp and 50bp NC1(MHC lanes), NC2(MHC lanes), NC3(MHC lanes), LC1(MHC lanes), ED(MHC lanes)

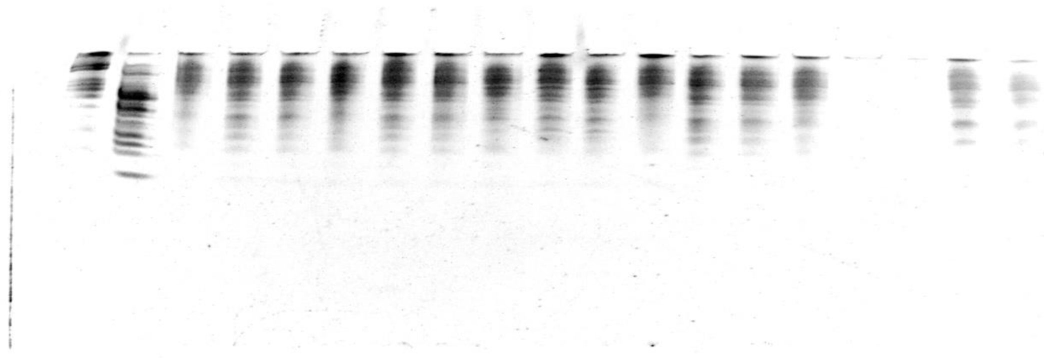

EcoR1-G+MH3-TCC combination; 100 bp and 50bp NC1(MHC lanes), NC2(MHC lanes), NC3(MHC lanes), LC1(MHC lanes), ED(MHC lanes)

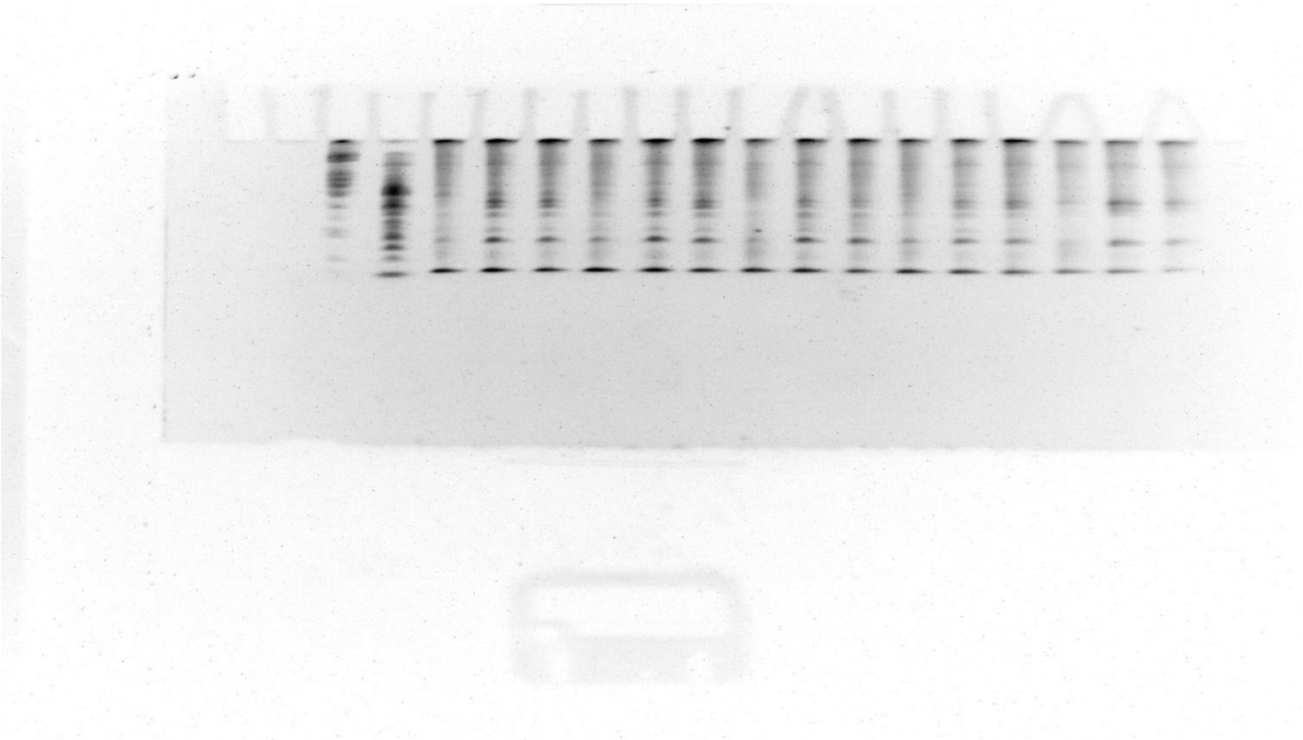

EcoR2-T+MH3-TCC combination; 100 bp and 50bp NC1(MHC lanes), NC2(MHC lanes), NC3(MHC lanes), LC1(MHC lanes), ED(MHC lanes)

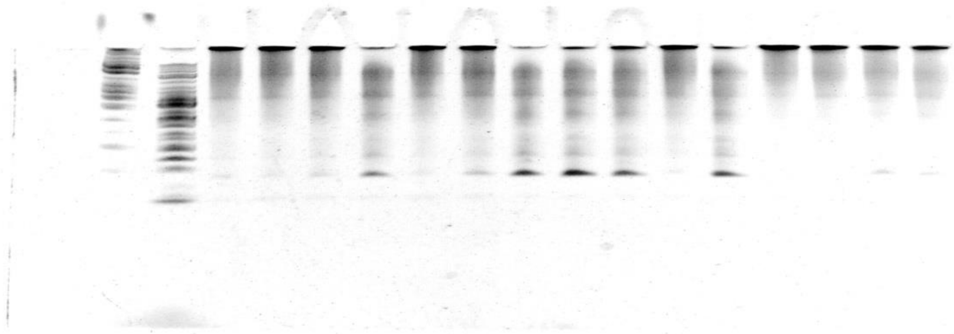

EcoR1-G+MH4-AAC C combination; 100 bp and 50bp NC1(MHC lanes), NC2(MHC lanes), NC3(MHC lanes), LC1(MHC lanes), ED(MHC lanes)

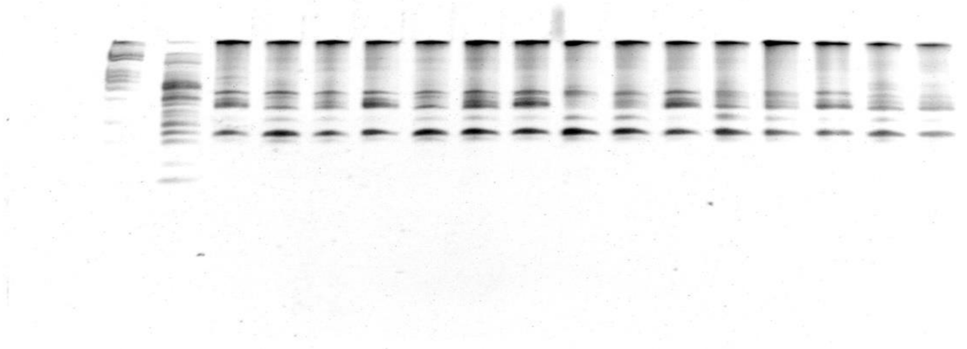

EcoR2-T+MH4-AAC C combination; 100 bp and 50bp NC1(MHC lanes), NC2(MHC lanes), NC3(MHC lanes), LC1(MHC lanes), ED(MHC lanes)

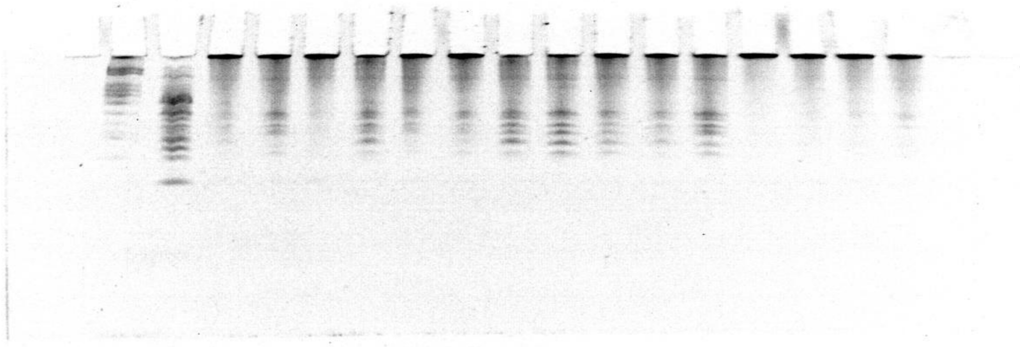

EcoR1-G+MH5-CGA A combination; 100 bp and 50bp NC1(MHC lanes), NC2(MHC lanes), NC3(MHC lanes), LC1(MHC lanes), ED(MHC lanes)

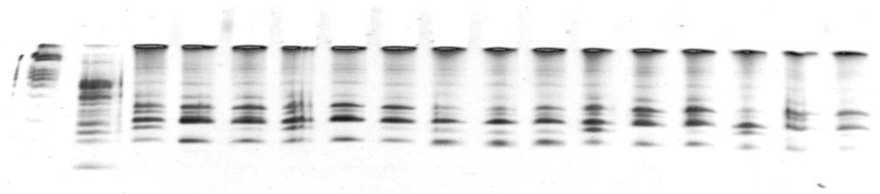

EcoR2-T+MH5-CGA A combination; 100 bp and 50bp NC1(MHC lanes), NC2(MHC lanes), NC3(MHC lanes), LC1(MHC lanes), ED(MHC lanes)

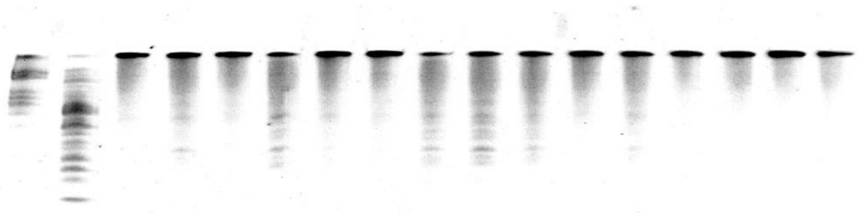

EcoR1-G+MH6-TAG C combination; 100 bp and 50bp NC1(MHC lanes), NC2(MHC lanes), NC3(MHC lanes), LC1(MHC lanes), ED(MHC lanes)

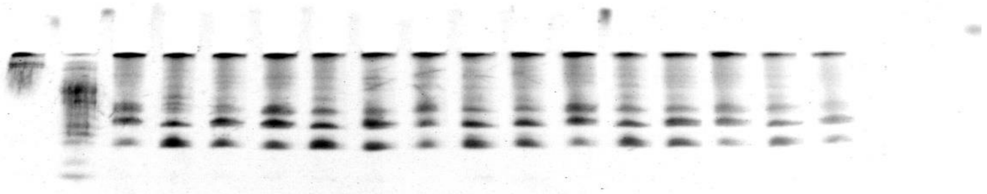

EcoR2-T+MH6-TAG C combination; 100 bp and 50bp NC1(MHC lanes), NC2(MHC lanes), NC3(MHC lanes), LC1(MHC lanes), ED(MHC lanes)

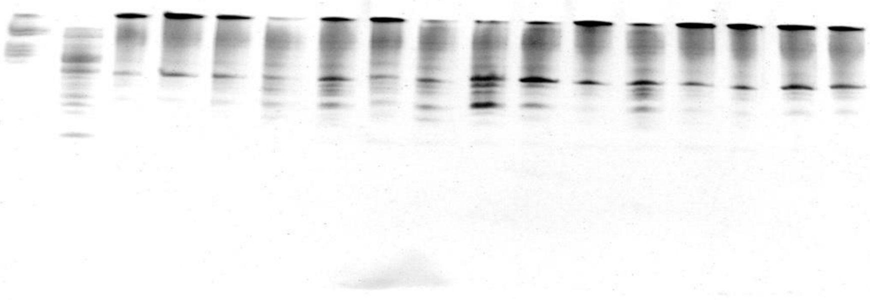

Supplement: Supplementary file 1 — Supplementary Information. [file 41598_2022_16530_MOESM1_ESM.pdf]
